# Stillbirths and the race-specific gap in neonatal death among extremely preterm births

**DOI:** 10.1038/s41598-025-22790-w

**Published:** 2025-11-06

**Authors:** Tim A. Bruckner, Allison Stolte, Brenda Bustos, Alison Gemmill, Joan A. Casey, Hedwig Lee, Ralph A. Catalano

**Affiliations:** 1 Department of Health, Society, and Behavior, Joe C. Wen School of Population and Public Health, University of California, Irvine, CA USA; 2 Center for Population, Inequality, and Policy, University of California, Irvine, CA USA; 3 Department of Population, Family, and Reproductive Health, Johns Hopkins Bloomberg School of Public Health, Baltimore, MD USA; 4 Department of Environmental and Occupational Health Sciences, University of Washington School of Public Health, Seattle, WA USA; 5 Department of Epidemiology, University of Washington School of Public Health, Seattle, WA USA; 6 Department of Sociology, Duke University, Durham, NC USA; 7 School of Public Health, University of California, Berkeley, CA USA; 8 Department of Sociology, University of Virginia, Charlottesville, VA, USA

**Keywords:** Stillbirth, Fetal death, Survival advantage, Extremely preterm births, Race differences, Neonatal death, Public health, Epidemiology

## Abstract

In the extremely preterm period (ePTB; less than 28 weeks), non-Hispanic (NH) Black infants in the US show relatively *lower* risk of neonatal death than do NH white infants. Explanations for this survival advantage include higher levels of stillbirth among NH Black persons, which could leave behind hardier members of the conception cohort that survive to birth. We test this “high stillbirth” explanation in the US and focus on NH Black singleton ePTB males given their large survival advantage. We applied time-series methods to 288 monthly conception cohorts (1995–2018 US fetal, birth, and neonatal death records) for NH Black and NH white singletons in the ePTB range (N = 473,472). We specified positive monthly outliers in male relative to female NH Black stillbirths in the ePTB range to gauge high NH Black male stillbirths. NH Black male ePTB singleton infants show a stronger neonatal survival advantage (relative to NH whites) for cohorts with high NH Black male stillbirth (4.4 fewer deaths per 100 live births, standard error = 1.3, p < .001). Cohort variation in fetal loss, as measured by high NH Black male stillbirth, may explain a portion of the counterintuitive racial/ethnic patterns in live birth mortality among extremely preterm births.

## Introduction

Infants born extremely preterm (ePTB; born at less than 28 weeks) comprise less than one percent of all live births but over 40% of all infant deaths^1,2^. Among those who survive past infancy, ePTB infants (relative to later-born infants) show greater risk of developmental delays and child morbidity^3–5^. In the US, Non-Hispanic (NH) Black infants show a greater incidence of ePTB relative to non-Hispanic (NH) white infants^6^. This greater incidence contributes substantially to the large, persistent, and well-described Black/white disparity in overall infant mortality in the US^7^. Explanations for this broader disparity include large set of social, economic and structural causes (including institutional racism)^8–10^.

Counterintuitively, NH Black infants at each gestational age (GA) in the preterm (i.e. < 37 weeks) period show a *lower* risk of morbidity and infant mortality than do NH white infants^11^. This survival advantage is largest at extremely early gestational ages^12^. Previous work theorizes that elevated pregnancy loss among NH Black gestations—especially during the second and third trimesters—could account for this NH Black survival advantage among live births^13,14^. Perinatal epidemiologists refer to this presumed elevated level of pregnancy loss as a form of left truncation bias^15–17^.

Left truncation refers to the non-random loss of cohort members before the observation of live birth. If, for instance, the large set of factors that produce greater incidence of ePTB among NH Black deliveries also produces greater risk of stillbirths (delivery at ≥ 20 weeks of gestation of a fetus that died in utero), then relatively more NH Black stillbirths (vs. NH white) may occur. Given that selection in utero disproportionately affects gestations considered less robust^18,19^, such left truncation via stillbirth could result in relatively hardier NH Black infants born in the ePTB live birth cohort. The fact that NH Black persons show a substantially elevated risk of stillbirth (relative to NH white persons) indirectly supports this argument^20–22^.

The notion that elevated stillbirths in a pregnancy cohort account for at least a portion of the live birth survival advantage of NH Black (vs. NH white) ePTB infants in that same cohort seems relatively straightforward. Empirical tests, however, remain sparse largely owing to the questionable completeness of US stillbirth data. A recent Stillbirth Working Group, convened by the Eunice Kennedy Shriver National Institute of Child Health and Human Development (NICHD) Council^23^, noted challenges with the consistency and quality of stillbirth data collection across states and municipalities. The many data collection barriers highlighted by the Working Group indicate that stillbirth counts in US vital statistics remain underreported. In addition, the completeness of reporting likely differs by geography, as well as by racial/ethnic and socioeconomic groups.

In this paper, we attempt to overcome the above limitations in two ways: (i.) by using an independent variable which we believe sensitively gauges the intensity of selection in utero via stillbirth, and (ii.) by employing a rigorous time-series study design of cohorts defined by estimated month of conception. Specifically, we use the monthly ratio of NH Black male to female stillbirths during the ePTB period in a conception cohort to approximate intensity of left truncation for NH Black ePTB males. We view this stillbirth sex ratio as a valid estimator of excess male stillbirths because we know of no evidence to suggest that the quality of stillbirth reporting in the US differs with respect to sex. Stillbirth sex ratios, moreover, rise following unambiguous population stressors^24,25^. This work, along with extensive literature which documents elevated male sensitivity *in utero*^18,19,26^, further supports the use of this variable to gauge variation over time in male stillbirth loss using the time-series design setting we employ here. The use of this sex ratio as an independent variable, moreover, precludes Type I error (spurious association) between left truncation and ePTB survival arising from errors in data quality or reporting of stillbirths that occur equally across sex.

We test whether unexpectedly high levels of selection in utero in a conception cohort (as measured by positive outliers in the stillbirth sex ratio) correspond with relatively low risk of neonatal death (i.e., less than 28 days) among NH Black ePTB males. We apply rigorous time-series methods to the universe of US data on stillbirth, ePTB live birth, and neonatal deaths over a 25-year period. We contribute to the literature by providing, to our knowledge, the first empirical test of whether variation over time in left truncation in conception cohorts, via stillbirth, accounts for at least of portion of the counterintuitive survival advantage of NH Black (relative to NH white) ePTB infants.

## Methods

### Variables and data

We use 1995–2019 US fetal death and cohort linked birth / infant death files from the National Center of Health Statistics (NCHS), Division of Vital Statistics for our analysis (which allows us to calculate monthly conception cohort information for Jan 1995 through Dec 2018). These data include information on all fetal deaths and live births—including information on infant death—occurring in the US during the study period. Researchers commonly use the cohort linked birth / infant death files for reports and studies on births and infant health but have only recently begun to take advantage of the fetal death files^27^. Some states appear to underreport fetal deaths in the in the early stillbirth period (about 20–27 weeks gestation)^28^. NCHS, however, has worked to ensure that the states use consistent definitions and reporting of fetal death over the past few decades and utilize these data in their own reports^29^. As such, NCHS considers fetal deaths occurring at or after 20 weeks gestation suitable for reporting and research purposes^27^.

We define our analytic population as singleton pregnancies ending—either via fetal loss or live birth—in the extremely preterm (ePTB) period, defined conventionally as 20 weeks 0/7 days to 27 weeks 6/7 days^30^. We exclude records missing gestational age for 2.25% of singleton fetal deaths (N missing = 12,950) and 0.52% of singleton live births (N missing = 503,601), as well as two records lacking plurality. We then further limit our analytic population to NH Black and NH white fetal deaths (NHB: N = 83,086; NHW: 116,145; N missing race-ethnicity = 5.79%) and live births (NHB: N = 198,511; NHW: 203,367; N missing race-ethnicity = 1.28%). We use the reported race and ethnicity of the birthing person recorded on the birth certificate.

All fetal deaths and live births in this final analytic population report sex, allowing us to later calculate stillbirth sex ratios and sex-specific infant death among all pregnancies ending in the ePTB period. Using information on infant deaths, we record a measure indicating neonatal death (death within the first 28 days of life = 1; and 0 otherwise).

Our analysis requires the aggregation of information on these fetal deaths, live births, and neonatal deaths across monthly conception cohorts for both NH Black and NH white male and female gestations. The NCHS data, however, do not include a variable on estimated date of conception. We, therefore, estimate date of conception by subtracting reported gestational age (in weeks) from the month and year of event (i.e., fetal death or birth). Absent information on exact date of event, we generated a pseudo “date” of event (to the day) by randomly assigning each fetal death and live birth a day (e.g., 1 to 31, 1 to 30, or 1 to 28, depending on the month) within their given month and year of fetal death/birth. Then, we subtract from this date the reported number of gestational days (gestational week*7) at the time of the event and aggregate the data to the conception month (i.e., MMYY). This aggregation smooths any small errors in the randomization process and ensures a non-zero count of stillbirths and neonatal deaths by race-ethnicity and sex in every time period. Supplemental tests find consistently high correlations (all Pearson correlation coefficients ≥ 0.88) in the estimated conception month across 100 runs of the randomization process, further suggesting that the final randomization of day process does not introduce bias.

To ensure complete information on births and deaths across all conception cohorts, we retain data from the January 1995 to December 2018 (i.e., 288 months) conception cohorts. Owing to the availability of data on fetal deaths and live births > 20 weeks gestation, the aggregated data here *approximate* conception cohorts but do not include the earliest pregnancy losses (including failures to implant), miscarriages, and elective abortions.

Using the aggregated data, we calculate our dependent variable: risk of neonatal death among NH Black ePTB male singletons. This variable measures the total number of deaths before 28 days among all NH Black liveborn ePTB male singletons divided by all such live births in a given conception month**.** For reasons described below, we also calculate this same measure for NH white male and NH Black female singleton ePTBs. Our independent variable—positive monthly outliers in the stillbirth sex ratio—gauges the relative excess of NH Black male stillbirths for a given conception cohort.

The UC Irvine Committee for the Protection of Human Subjects approved this study (protocol # 20195444). All methods conform to the guidelines and regulations set forth in the study protocol. No personal identifiers are included in our analysis.

Analysis. Testing our hypothesis required that we identify monthly conception cohorts exhibiting unexpectedly high counts of early (i.e., before 28 complete weeks of gestation) NH Black male stillbirths in the US over our test period. As described below, we specified these “high male stillbirth” cohorts as those with NH Black male to female ratios of stillbirth above the 99.5% confidence interval (CI) of values expected from the entire study period. These high male stillbirth cohorts serve as our independent variable.

We specified the outcome variable as the difference between NH white male and NH Black male rates (i.e., risk difference) of neonatal death among ePTB singletons. Use of this difference as the outcome variable makes explicit the use of NH white males as a comparison group, which we assume would remain unaffected by high NH Black male stillbirths in that conception cohort. This “difference in difference” approach “controls” confounders that affect neonatal death among both NH Black and NH White extremely preterm male infants. These confounders include shared trends and seasonality (i.e., autocorrelation) as well as “exogenous shocks” such as weather extremes, pandemics, or improvements in the efficacy of medical care. Equally important, this approach allows us to estimate the extent to which the Black/White “gap” in neonatal survival responds to unusually high NH Black male stillbirths. We hypothesize that this difference will increase during months of high NH Black male stillbirths (i.e., more left truncation would translate to fewer frail NH Black ePTB infants compared to other conception cohorts, and therefore a greater Black neonatal survival advantage relative to white males).

Our test proceeded through the following steps.

1. We defined “high male stillbirth” cohorts as those with NH Black male to female ratios of early stillbirth above the 99.5% CI of values expected from history. We did this by estimating the expected value and 99.5% CI of the ratios for the 288 monthly conception cohorts over the test period. We could not use the mean of the cohort ratios as their expected value because the ratios were not independent of each other (i.e., they exhibited autocorrelation). We, therefore, used Box-Jenkins^31^ methods to identify and model autocorrelation in the series. The fitted values of the Box-Jenkins model exhibiting the lowest Akaike Information Criterion (AIC) served as expected values. The model residuals gauged the degree to which the observed ratios of male to female early stillbirths differed from expected. These residuals exhibit no autocorrelation, have a mean of 0, and a 99.5% CI equal to 2.83 times their standard deviation (SD).
2. We applied the methods of Chang, Tiao, and Chen^32^ to detect residuals outside the 99.5% CI. We specified monthly cohorts with residual values above the CI as our high male stillbirth cases.
3. In a separate model, we used the methods of Box and Jenkins^31^ to identify autocorrelation in the difference in neonatal death between NH white male and NH Black male ePTB singletons. If the series exhibited autocorrelation, we built the best fitting (i.e., lowest AIC) Box-Jenkins model of the series to arrive at values expected from history.
4. We tested our hypothesis by estimating coefficients in an equation formed by expanding the model built in Step 3 to include a variable scored 1 for the high male stillbirth cohorts (identified in Step 2) and 0 otherwise. We inferred support for the hypothesis if the coefficient for the NH Black high male stillbirth variable was above its 95% CI (i.e., positively signed and more than 1.96 times its standard error [SE]).

## Results

**Fig. 1:**
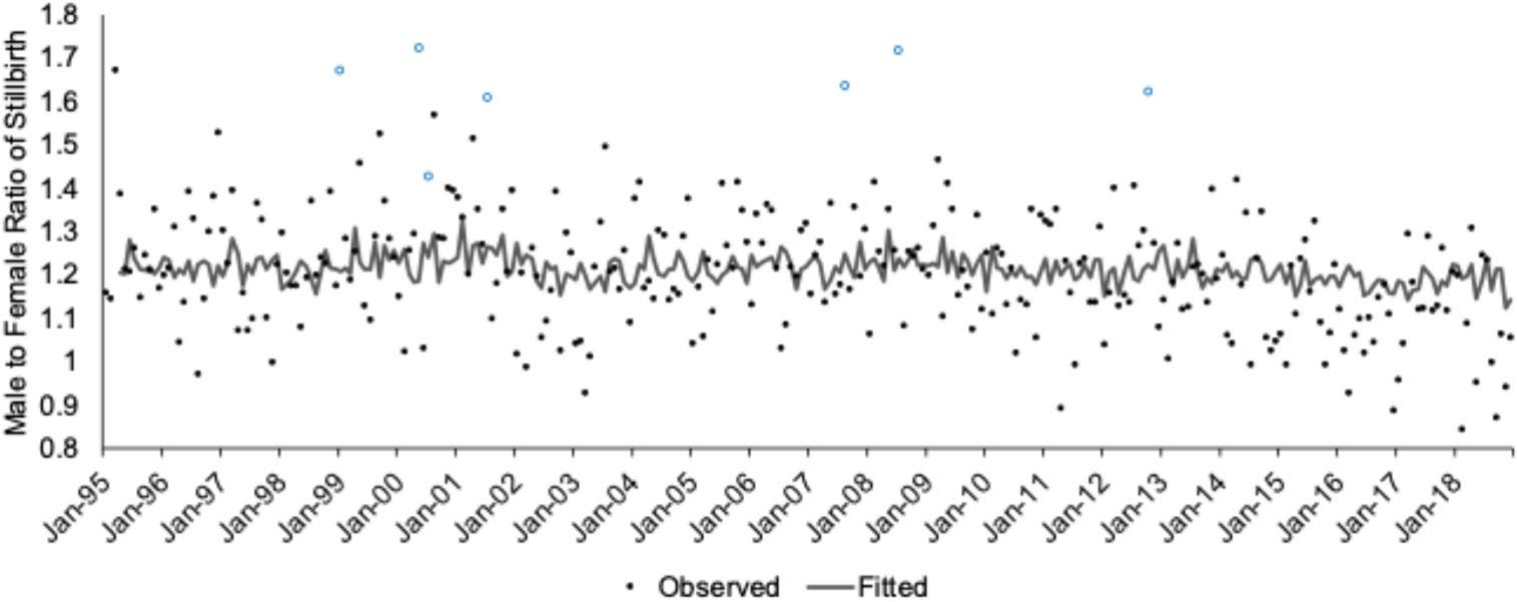
Observed (dots) and fitted (line) values of the NH Black stillbirth male to female sex ratio across 288 monthly conception cohorts, January 1995 to December 2018. Positive outliers shown in **BLUE.**

**Fig. 2:**
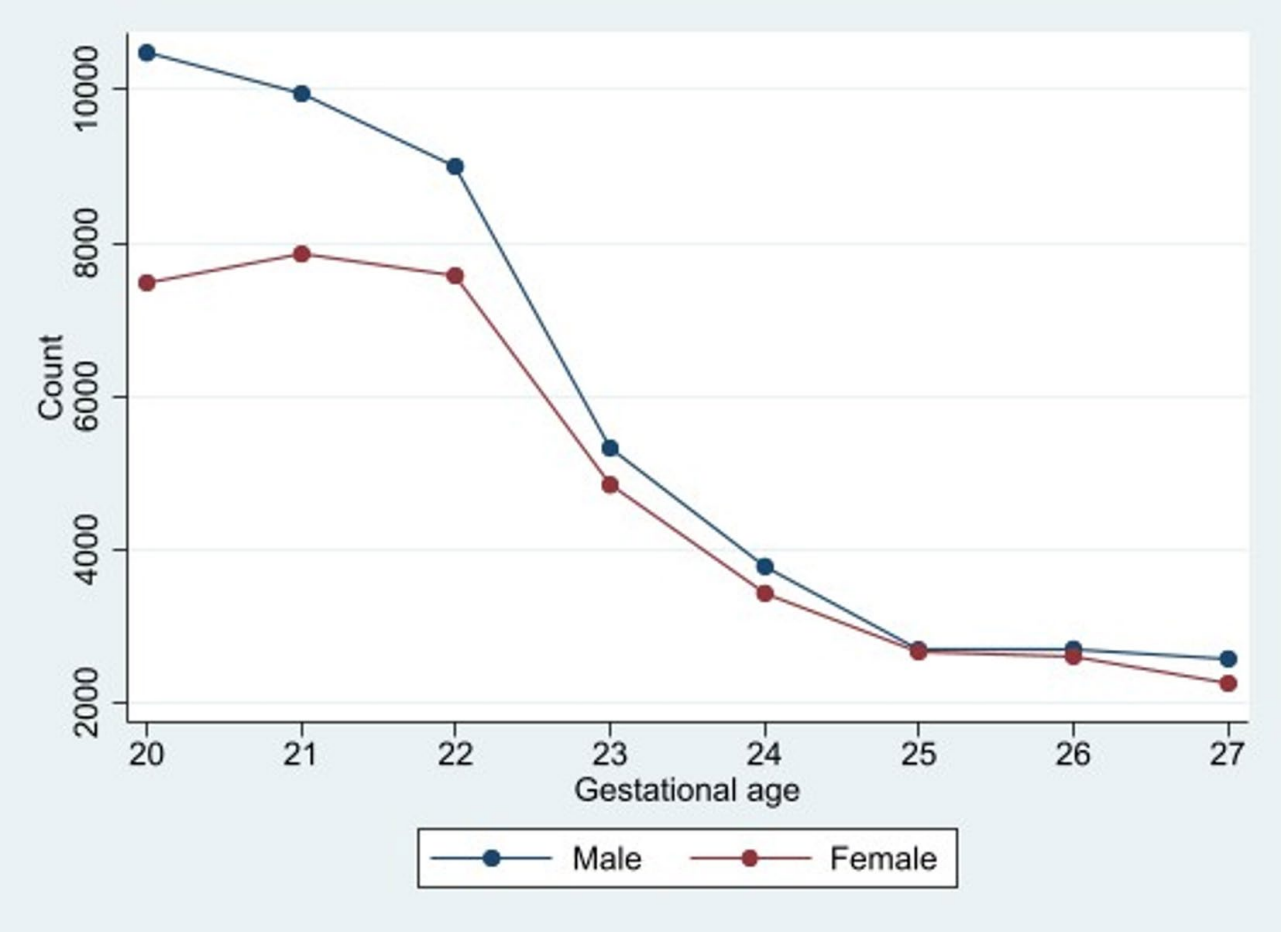
Histogram of count of NH Black stillbirths, by sex, across ePTB gestational age range, over the entire test period.

We include over 88,000 early (20 to < 28 weeks gestation) NH Black stillbirths in the US over the test period (Table 1). The NH Black male to female ratio of early stillbirths ranged from 0.845 to 1.722. Consistent with prior work, the monthly mean ratio shows a male skew (mean: 1.214; SD 0.1476). Figure 1 shows the NH Black stillbirth sex ratios, as circles, plotted over time. To give the reader a sense of the recorded gestational age of stillbirths over the ePTB period, Fig. 2 plots the histogram of NH Black stillbirths, by sex, across gestational age.

**Table 1:** Summary statistics of NH Black and NH white extremely preterm births (ePTBs) among 288 monthly conceptions between 1995 to 2018.

	Total N	Monthly Mean	Monthly SD	Range
NH Black				
ePTB only				
Male live births	99,058	343.95	36.85	240 to 474
Neonatal deaths	32,014	111.16	19.67	61 to 154
Female live births	91,578	317.98	31.10	235 to 405
Neonatal deaths	26,274	91.23	15.81	54 to 134
Early stillbirth only				
Male	48,226	167.45	20.41	109 to 229
Female	39,940	138.68	14.93	94 to 179
NH White				
ePTB only				
Male live births	105,278	365.55	35.99	260 to 439
Neonatal deaths	35,983	124.94	21.95	77 to 175
Female live births	90,501	314.24	30.02	241 to 402
Neonatal deaths	28,463	98.83	17.68	51 to 151

ePTB measures all live births occurring between 20 to < 28 weeks gestation; early stillbirth measures all fetal deaths occurring between 20 to < 28 weeks gestation.

**Fig. 3:**
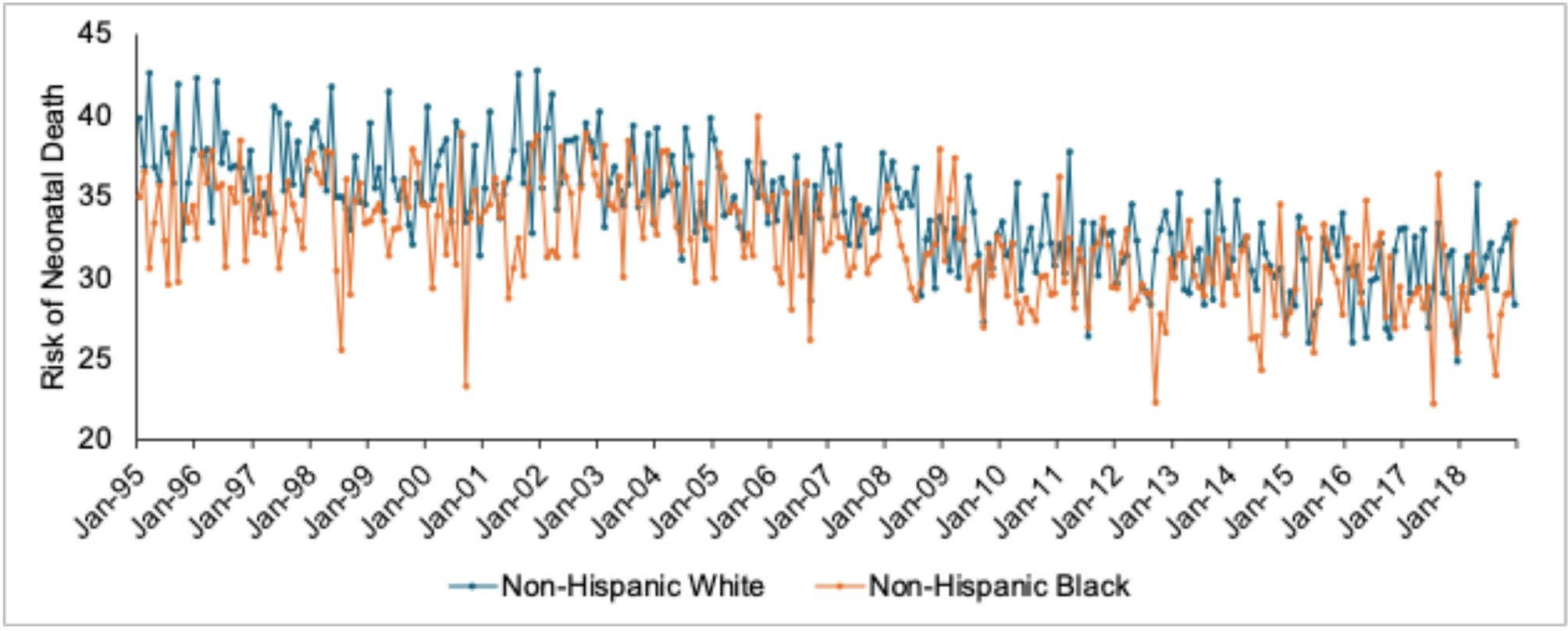
Risk of neonatal death per 100 births among NH white (Blue) and NH Black (Orange) male singletons born extremely preterm among 288 monthly conceptions, 1995 to 2018.

**Fig. 4:**
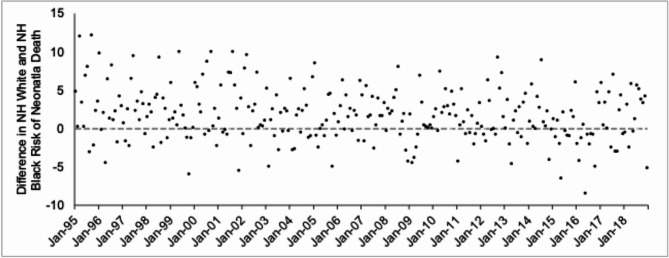
Difference in the risk of neonatal death per 100 births among NH white and NH Black extremely preterm male singletons among 288 monthly conceptions, 1995 to 2018.

Of the over 190,000 ePTB singleton NH Black live births in the test period, over 58,288 (i.e., 30.5 per 100) ended in a neonatal death (Table 1). The risk of neonatal death among ePTB NH Black male and NH white male singletons shows substantial monthly variation across conception cohorts and declines over time (Fig. 3). Figure 4 plots our key dependent variable—that is, the NH white-NH Black risk difference in neonatal death among ePTB male singletons. Consistent with prior literature, the positively signed mean indicates, on average, an excess risk of neonatal death among NH white males (i.e., NH Black survival advantage).

In *Step* 1 of our test, we detected and modeled autocorrelation in the NH Black male to female ratio of early stillbirths. The best fitting model for the series was as follows in which all the estimated parameters are at least twice their standard errors.

1 $${\text{Z}}_{{{\text{1t}}}} = {1}.{212} + \left( {{1} + 0.{\text{161B}}^{{9}} } \right)/\left( {{1} - 0.{\text{148B}}^{{3}} } \right){\text{a}}_{{\text{t}}}$$

Z_1t_ is the observed NH Black male to female ratio of early stillbirths in a given conception month t. 1.212 is a constant (standard error [SE] = 0.001).

B^n^ are backshift operators or the value of a_t_ at months t-9 and t-3. − 0.161 is a moving average parameter (at lag 9 months) and 0.148 is an autoregressive parameter (at lag 3 months).

0.161 (SE = 0.06) is a moving average coefficient indicating that high or low values of Z_1t_ (i.e., NHB ePTB stillbirth sex ratio) at month t echo nine months later.

0.148 (SE = 0.06) is an autoregressive coefficient indicating that high or low values of Z_1t_ (i.e., NHB ePTB stillbirth sex ratio) at month t repeat, although in diminishing amounts, three months later.

a_t_ is the model residual, or difference between expected and observed values of Z_1t_, at time t. These residuals have a mean of 0, a standard deviation of 0.142, and exhibit no autocorrelation (i.e., are statistically independent of each other).

Figure 1 shows, as a line, the fitted values of the above model. These serve as values expected from history.

In *Step* 2, we used outlier detection routines to identify our “high male stillbirth” cohorts based on model residuals, which are the differences between the line and points shown in Fig. 1. These routines detected seven high outliers: Jan-1999, May-2000, July-2000, July-2001, Aug-2007, Jul-2008, and Oct-2012. These cohorts with residuals greater than 0.31 (i.e., those above the 99.5% CI) are shown with **blue circles** in Fig. 1.

*Step* 3 specified the dependent variable as the difference between NH white male and NH Black male rates of neonatal death among ePTB singletons. We found no autocorrelation in the series. We, therefore, estimated a regression equation (Step 4) in which this ratio was modeled as function of a constant and a variable scored 1 for the high male stillbirth cohorts identified in Step 2, and 0 otherwise. The results of that estimation were as follows.

2 $${\text{Z}}_{{{\text{2t}}}} = 0.0{17} + 0.0{\text{44X}}_{{\text{t}}} + {\text{a}}_{{\text{t}}}$$

Z_2t_ is the difference between NH white male and NH Black male rates of neonatal death among ePTB singletons in a given conception month t. Here, 0.017 (SE = 0.002) is the mean of Z_2_ which implies that, among ePTB singletons, NH white males show, on average, a 1.7 per 100 live birth excess risk of neonatal death in a conception month relative to NH Black males. We inferred support for our hypothesis because the coefficient for the high male stillbirth cohorts X_t_ (i.e., 0.0442) was positively signed and more than 1.96 times its standard error (i.e., 0.0131, T-value = 3.38, p < 0.001). This coefficient implies an increased NH white / NH Black neonatal survival gap of 4.42 per 100 ePTB births in these seven conception cohorts. This value substantially increases the neonatal survival advantage of NH Black ePTB males relative to NH white ePTB males (i.e., for these seven cohorts, the risk difference moves from a mean of 1.7 excess neonatal deaths per 100 ePTB among NH white males to a 6.1 excess neonatal deaths per 100 NH white ePTB males).

Our test arises from the argument that selection in utero contributes to the unexpectedly low rate of neonatal death among NH Black extremely preterm males compared to the rate among their NH White counterparts. But that argument implies another prediction regarding a second, perhaps less provocative, gap in neonatal death – that between NH Black male and female infants born extremely preterm. Here males appear at a disadvantage in that they exhibit higher rates than females. If our theory is robust, that gap should appear relatively small in the high male stillbirth cohorts. We test that prediction by repeating steps 3 and 4 above but instead with a dependent variable specified as the difference between NH Black male and NH Black *female* rates of neonatal death among ePTB singletons (i.e., risk difference). Here, the interpretation of the high stillbirth sex ratio coefficient would relate to the difference in neonatal survival between NH Black male and NH Black female ePTB infants.

**Fig. 5:**
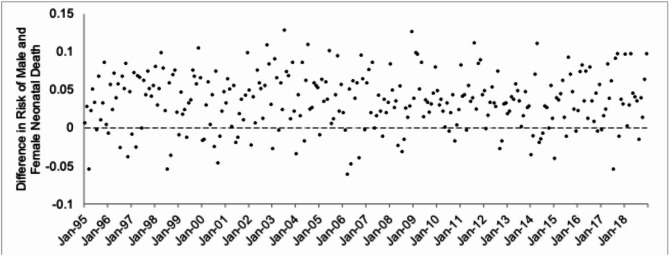
Difference in risk of NH Black extremely preterm male and female neonatal deaths per 100 births among 288 monthly conceptions, 1995 to 2018.

In the robustness check, the dependent variable (i.e., risk difference of neonatal death between NH Black ePTB male and female singletons) ranged from -0.062 to 0.129 with a mean of 0.036 (SD = 0.037). Figure 5 shows, as circles, the risk differences plotted over time. We detected no autocorrelation in this series, which then led us to directly expand the equation to include a variable scored 1 for the seven high male stillbirth cohorts identified in Step 2, and 0 otherwise. The results of that estimation were as follows.

3 $${\text{Z}}_{{{\text{3t}}}} = 0.0{37} - 0.0{\text{465X}}_{{\text{t}}} + {\text{a}}_{{\text{t}}}$$

Here, Z_3t_ is the risk difference (i.e., male minus female) of neonatal death among NH Black ePTB singletons. 0.037 (SE: 0.0022) is the mean of Z_3t_ which implies that among ePTB singletons born during our test period, NH Black males show a 3.7 per 100 ePTB births excess risk of neonatal death when compared to NH Black female ePTB. a_t_ is the model residual (i.e., error term) for the cohort conceived in month t. X_t_ is the variable scored 1 for the high-stillbirth cohorts identified in Step 2 and 0 otherwise. We inferred support for the hypothesis because the coefficient for the high-stillbirth variable (i.e., − 0.0465) was negatively signed and more than 1.96 times its standard error (SE: 0.014, T = − 3.35, p < 0.001). This coefficient indicates that the male to female excess risk of neonatal death among NH Black ePTB infants in the high male stillbirth cohorts was 4.65 per 100 ePTB *lower* than expected. Put another way, in these high stillbirth cohorts, NH Black males born ePTB would show an absolute risk of neonatal death that falls below the baseline risk of NH Black females born ePTB.

Whereas our tests use high positive outliers in the NH Black stillbirth sex ratio as the independent variable, it remains possible that more routine deviations in the stillbirth sex ratio may also correlate with the risk difference in ePTB neonatal death. We therefore empirically assessed the contribution of the continuous stillbirth sex ratio series—net of autocorrelation and the positive outliers we detected— to the outcome. This continuous independent variable series consists of the residual values of the NH Black stillbirth sex ratio after time-series removal of autocorrelation and removal of the detected positive monthly outliers (p < 0.005) based on routines devised by Chang, Tiao, and Chen^32^. Time-series results, in which we also included this continuous series as an independent variable, show no relation between these continuous residuals and risk of neonatal death among NH Black ePTB males (coef: −0.0149, SE: 0.017, p = 0.38). The null result for the continuous series indicates that routine deviations in the stillbirth sex ratio do not correlate with that cohort’s risk of neonatal death among ePTB births. The positive conception cohort “outlier” variable, by contrast, remains strongly correlated with the outcome (coef: 0.044, SE: 0.0129, p < 0.0001), indicating (as with the original test) a larger than expected NH White minus NH Black gap in ePTB neonatal death risk during months of very high male stillbirth.

## Discussion

At each gestational age before term, NH Black infants show a neonatal survival advantage relative to NH whites^12^. This survival advantage appears larger in the ePTB period compared to after 28 weeks of gestation. Previous work suggests^17,33–35^, but does not directly test, that high levels of NH Black stillbirth in a conception cohort could induce left truncation and thereby account for a portion of this live birth survival advantage. We test this “high stillbirth” explanation using US data over a 25-year time span and focus our analysis on NH Black male births. Findings support this explanation in that the risk of neonatal death among NH Black ePTB males falls in cohorts with unexpectedly high NH Black male stillbirths. Results, which remain robust to alternative specifications, indicate that selection in utero against stillbirths may account for a portion of the counterintuitive NH Black infant survival advantage especially in the ePTB period.

These findings further the line of research demonstrating the consequences of selection in utero for population-level infant health. Previous work, for example, points to twin fetal loss as an unmeasured confounder that shapes overall infant mortality rates^36,37^ among singleton births. Related work, moreover, suggests that higher fetal loss among NH Black pregnancies may explain the relative frailty of NH white (v. NH Black) ePTB singleton births in terms of birthweight^38^. Still, while these and our current findings suggest that at least part of the NH Black survival advantage may arise from higher selection in utero among NH Black pregnancies, a substantial, and paradoxical, gap remains in even non-outlier cohorts in our US data (Fig. 3).

Strengths of our study include the use of ePTB data for all NH white and NH Black births in the US over a 25-year period, which promotes population-based inference about this neonatal survival gap. We also align fetal death, ePTB live birth, and neonatal death data in a conception cohort framework to estimate the extent to which excess fetal deaths could signal left-truncation in live birth cohorts. Our rigorous time-series methods and use of stillbirth sex ratios as the independent variable, moreover, minimize threats that temporal patterning in neonatal deaths, unmeasured confounding, and/or measurement challenges of fetal death counts could drive our results.

This paper includes several limitations. First, neither the fetal death nor birth data include the true month of conception. Instead, we estimated conception cohorts using the month/year and gestational age at the time of event (either fetal death or live birth). Although this estimation process may produce small, random errors in the assigned conception dates, our aggregation to month minimizes concerns that this randomization process drives results. Second, although the fetal death files theoretically contain information on all fetal deaths occurring ≥ 20 weeks gestation in the US, prior work highlights the potential for misreporting^27^ or underreporting of fetal deaths, particularly in the extremely preterm period^28^. Our use of time series analysis and stillbirth sex ratios, however, assuage concerns that systemic underreporting or misreporting drive our results. The time-series models leverage deviation from temporally patterned means so that results remain unaffected by reporting discrepancies that appear stable over time. The stillbirth sex ratio, moreover, “absorbs” time sensitive changes in reporting that affect both NH Black male and female fetal deaths similarly.

Whereas time-series models allowed us to account for a large set of confounders, time-varying factors unique to NH Black males (e.g., healthcare access or quality, environmental exposures) could also affect cohort left truncation and the risk of neonatal death among ePTB infants. Future work may wish to leverage such individual-level data to assess the relative contribution of these factors.

We also note that, while use of high outliers in the stillbirth sex ratio does not eliminate underreporting bias entirely, it allows the detection of large anomalies that may reflect selective in utero loss. In addition, use of this independent variable means that we caution against using our results to estimate how smaller changes in selection in utero—changes that fall within the ‘expected’ range—may contribute to the NH Black survival advantage. Indeed, the additional analyses we performed, in which we separately evaluated the potential association between “routine” variations in stillbirth sex ratios and neonatal death among ePTB, showed no relation. We speculate that this circumstance may arise due to the stochastic “noise” or measurement error inherent in stillbirth sex ratios when using vital statistics data, or due to the fact that only larger shocks sufficiently perturb the left truncation process in a detectable way that affects that cohort’s risk of neonatal death. Distinguishing among these, and other, explanations for the null result of the continuous stillbirth sex ratio finding would benefit from ongoing improvements in quality and completeness of data collection on stillbirths.

Owing to limitations of vital statistics data, we did not have longitudinal individual-level information on pregnancies. For this reason, we could not identify detailed clinical characteristics of stillbirths. Having such rich clinical information, in future datasets, may better characterize the subgroups for whom the force of left truncation is most intense, as well as the types of neonatal death that may be “conserved” owing to cohort left truncation. We presume that such data may be available from specific medical centers or private health insurance groups, which could provide additional scientific rigor on understanding causal antecedents of stillbirth.

Work not focused on selection in utero offers additional speculation for this survival gap. For example, epidemiologic literature theorizes that collider/stratification biases emerge when selecting on the earliest gestations^39–41^. These papers reason that (1) some social characteristic X (here: race-ethnicity) predicts risks of early birth and mortality, (2) some unmeasured variable Z also predicts risks of both early birth and infant mortality, and, (3) if unmeasured variable Z more strongly predicts mortality than does social characteristic X, then focusing on outcomes among early births only without accounting for Z will distort the relationship between X (e.g., race-ethnicity) and mortality. In practice, this hypothetical circumstance would translate to NH white infants born in the extremely preterm period having a higher proportion of infants with some set of ummeasured variables Z than would NH Black infants. A result of this imbalance of Z across race/ethnicity, in this example, would lead to NH white infants appearing more frail and at risk of mortality than NH Black infants born during the same period. Whereas other descriptive work supports the plausibility of this explanation^39^, empirical tests of Z remain challenging given that it (by definition) represents unmeasured variables. As it relates to our specific test, we await more work in this area that develops predictions about the time-varying “dose” of Z in conception cohorts with varying levels of selection in utero and its implications for the NH Black survival advantage.

A related argument in the literature contends that greater exposure to racialized stressors may lead to an earlier “target” gestational age of delivery and downwardly shift the distribution of gestational ages for NH Black (v. NH white) births^39,42^. This left-shift in target gestational age, presumably without any detrimental effect on fetal development, would lead to a circumstance in which NH Black infants appear intrinsically less frail especially at early gestational weeks. Studies that use a fetuses-at-risk approach—which adjusts for the greater proportion of NH Black births in the earliest periods of viable gestation—demonstrate a reversal of the NH Black survival advantage^42^. This work, however, has yet to identify putative causes of an earlier target GA in particular subgroups and does not invalidate the important clinical fact of a lower risk of neonatal death of NH Black (v. NH white) ePTB infants in the US.

Importantly, structural and interpersonal forms of racism likely contribute to the above-described higher fetal losses and higher selection into extreme preterm for NH Black pregnancies^43^. Previous work emphasizes that structural factors such as inequitable socioeconomic, political, criminal justice, and environmental contexts contribute to worse health among minoritized racial-ethnic groups by disproportionately exposing those groups to health-deteriorating stressors (e.g., state-sanctioned violence, environmental toxins) and creating and maintaining barriers to quality health care and other relevant resources (e.g., education, employment, and housing)^44,45^. Parallel studies emphasize that lifelong exposure to racial discrimination accelerates biological aging^46,47^. These health-deteriorating contexts and experiences, even if experienced prior to pregnancy—can pass on health disadvantages to one’s offspring via the in utero environment and metabolic and physiological changes that increase risks related to fetal development^47,48^. During pregnancy, exposure to racism continues to shape perinatal outcomes by, for example, shaping Black birthing persons’ utilization and quality of prenatal care^49,50^—important for monitoring and supporting fetal development—and exposing Black birthing persons to micro-aggressions^49^ that predict early birth. Although less-studied, these same processes likely result in higher fetal loss among NH Black pregnancies, with prior work demonstrating that in utero selection^51^ may respond to changing social environments. We encourage future studies to more directly measure the association between racialized inequities and selection into fetal loss and extremely early birth, as well as the mechanisms that drive those relationships.

The NICHD-convened Stillbirth Working Group, which investigated the quality and reporting of fetal death data in the US, identified several impediments to complete case reporting^23^. Hospitals with a lower volume of births may have less resources to fill out fetal death reports to the state. In addition, less-resourced hospitals may not have the staff to perform an autopsy, or may have difficulties ascertaining the sex of the fetal death^27^. Furthermore, Medicaid insurance does not cover autopsy costs, which limits completeness of fetal death data for those with publicly-funded health insurance^52^. These circumstances imply that underreporting and/or incomplete reporting of fetal death data may disproportionately occur in lower-income regions and/or lower SES pregnant persons. We, however, know of no research in the US which has rigorously examined the potential of systematic bias in fetal death reporting; we encourage such important work in the future.

The Stillbirth Working Group also suggested several areas of improvement^23^. Some of these areas include more complete ascertainment of events^53^, greater precision of gestational age of event, and autopsy to determine cause of fetal death. We agree that improved data collection protocols^54,55^, and adherence to such protocols, holds the potential for researchers and clinicians to further characterize, and potentially redress, the causes of stillbirths. Furthermore, we contend that improvements in data quality may help with detecting even more subtle signals of selection in utero that could assist clinicians with predicting morbidity and mortality among live births born in the ePTB period—of which a disproportionate share are NH Black births.

## Data Availability

All de-identified data will be made publicly available on our GitHub repository, https://github.com/vazqueb3/ePTB_stillbirths_bwdis.git.
